# The Mechanics of Mitotic Cell Rounding

**DOI:** 10.3389/fcell.2020.00687

**Published:** 2020-08-06

**Authors:** Anna V. Taubenberger, Buzz Baum, Helen K. Matthews

**Affiliations:** ^1^Biotechnology Center, Center for Molecular and Cellular Bioengineering, Technische Universität Dresden, Dresden, Germany; ^2^MRC Laboratory for Molecular Cell Biology, University College London, London, United Kingdom

**Keywords:** mitosis, mitotic rounding, myosin, ezrin, Ect2, actin cortex, osmotic pressure, cell mechanics

## Abstract

When animal cells enter mitosis, they round up to become spherical. This shape change is accompanied by changes in mechanical properties. Multiple studies using different measurement methods have revealed that cell surface tension, intracellular pressure and cortical stiffness increase upon entry into mitosis. These cell-scale, biophysical changes are driven by alterations in the composition and architecture of the contractile acto-myosin cortex together with osmotic swelling and enable a mitotic cell to exert force against the environment. When the ability of cells to round is limited, for example by physical confinement, cells suffer severe defects in spindle assembly and cell division. The requirement to push against the environment to create space for spindle formation is especially important for cells dividing in tissues. Here we summarize the evidence and the tools used to show that cells exert rounding forces in mitosis *in vitro* and *in vivo*, review the molecular basis for this force generation and discuss its function for ensuring successful cell division in single cells and for cells dividing in normal or diseased tissues.

## Introduction

Cell division requires the separation and equal partition of DNA and cellular contents into two daughter cells. To achieve this, animal cells undergo a remarkable series of structural changes when they enter mitosis, which impact every cellular compartment. Chromosomes condense and enter the cytoplasm following loss of nuclear envelope integrity. Centrosomes separate, and microtubules form a bipolar spindle to array chromosomes at metaphase before pulling them apart to segregate the genetic material at anaphase. However, mitosis is accompanied by an equally dramatic series of morphological changes (reviewed in Ramkumar and Baum, 2016). In adherent cells, these begin in early prophase when cells decrease substrate adhesion and round up to assume a characteristic spherical shape. This process of cell rounding in early mitosis is a near universal feature of animal cell division and is observed widely in many cell types in 2D and 3D culture as well as in tissues. It has recently become appreciated that mitotic rounding also plays an important role in facilitating successful cell division. By creating a spherical cell shape at metaphase, mitotic rounding ensures that there is sufficient space within the cell to form a mitotic spindle (reviewed in Cadart et al., 2014).

Changes in cell shape require force. In this review, we focus on the biophysical changes that generate the forces required for mitotic rounding. We discuss how the forces generated by the acto-myosin cytoskeleton change cell mechanics and act, along with loss of adhesion and changes in intracellular pressure, to drive mitotic rounding. And we explore the function of mitotic rounding in enabling cells to exert force against their environment to aid cell division in normal tissues and in diseases associated with stiffened tissue.

## Forces Accompanying Entry Into Mitosis

**FIGURE 1:**
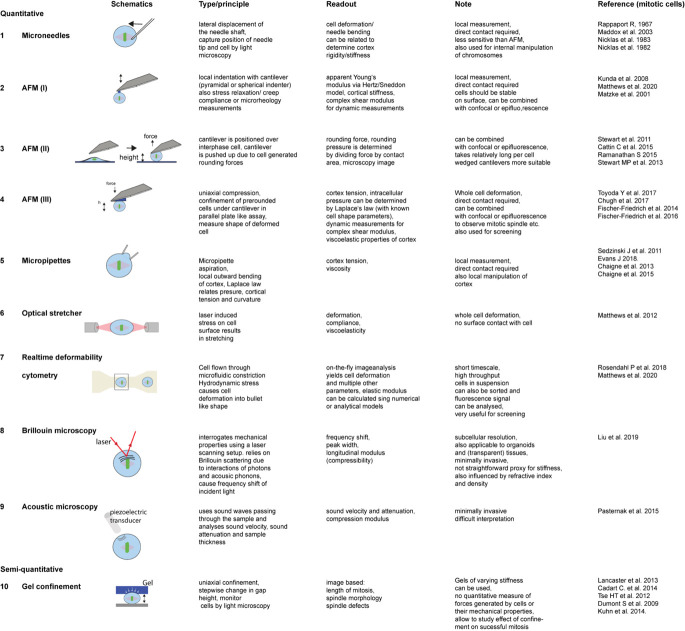
Techniques that have been applied to measure mitotic cell mechanics. For detailed description of methods (see Box 1).

Adherent cells grown on an artificial substrate are usually spread flat in interphase but round up to become spherical at mitotic entry. Part of this shape change is caused by loss of adhesion to the substrate. The classical integrin-containing focal adhesion complexes that anchor cells to the extracellular matrix (ECM) are disassembled at mitotic entry (Dao et al., 2009; Dix et al., 2018) and cells remain loosely attached by beta1-integrin containing retraction fibers (Cramer and Mitchison, 1997; Dix et al., 2018) and atypical adhesions under the cell body (Lock et al., 2018). However, mitotic rounding is not simply induced by loss of adhesion causing the cell to adopt a spherical shape like a liquid droplet due to its surface tension. Many studies have demonstrated that it is, instead, a process involving the active generation of forces. Multiple different techniques have been applied to measure the forces associated with mitotic rounding, which are summarized in Figure 1 and described in detail in Box 1. Mechanical terms used throughout this review are defined in the Glossary.

**Box 1. Techniques to measure cell mechanical properties and force generation during mitotic rounding.**

Over the past decades, various techniques have been employed to characterize cell mechanical properties or quantitate forces generated by cells (reviewed by Moeendarbary and Harris, 2014; Wu et al., 2018) and many have been applied to probe mitotic cells or to mechanically manipulate them. These assays classically apply local or global stress onto a cell and study the resulting deformation response to characterize the elastic or viscoelastic properties of the cell (Wu et al., 2018). A simple assay makes use of microneedles to poke cells (Figure 1.1; Rappaport, 1967; McConnaughey and Petersen, 1980; Maddox and Burridge, 2003). Thereby the shaft of a calibrated needle is laterally pushed against a cell by a certain distance and the resultant deflection of the needle tip is measured. Itabashi et al. (2012) used a pair of vertically oriented microfabricated cantilevers to exert mechanical impulses on mitotic allowing for cell manipulation at various directions with respect to the metaphase plate. A more precise force readout and manipulation is enabled by **atomic force microscopy (AFM)** (Binnig et al., 1986), which has become the gold standard for the mechanical characterization of cells and tissue (Figure 1.2–1.4; Krieg et al., 2019). In the simplest application, a cantilever is lowered at defined speed onto a cell through piezo elements while reading out at the same time the resultant force acting on the cantilever, from which a force-distance curve is obtained (Figure 1.2). The force-indentation relationship can also be characterized in a time-dependent manner, e.g., by stress relaxation, creep compliance or oscillatory measurements. Depending on the cell mechanical model (e.g., elastic solid, liquid droplet, poroelastic body), type of indentation (static, dynamic), different mechanical parameters can be obtained, such as apparent Young’s moduli (Radmacher et al., 1996; Matzke et al., 2001), surface tension and/or intracellular pressure (Rosenbluth et al., 2006; Krieg et al., 2008; Cartagena-Rivera et al., 2016), a poroelastic diffusion constant (Moeendarbary et al., 2013), and shear storage and loss moduli (Alcaraz et al., 2003; Rother et al., 2014; Schierbaum et al., 2015). Moreover, cell generated forces can be measured at the onset of mitotic rounding and along mitotic progression (Stewart et al., 2011a; Figure 1.3), where a more stable confinement can be facilitated by the use of wedged (Stewart et al., 2013) or microfabricated (Cattin et al., 2015) cantilevers (Figure 1.4). AFM enabled uniaxial confinement has also been combined with confocal microscopy to accurately determine the 3D contour of the deformed cell, which allows to calculate the associated pressure and surface tension according to the law of Laplace (Figure 1.4; Stewart et al., 2012; Fischer-Friedrich et al., 2014; Ramanathan et al., 2015; Chugh et al., 2017; Toyoda et al., 2017). To obtain a more detailed description of the rheological properties the cortex, the parallel plate assay can be modified by oscillating the wedged cantilever at pre-set frequencies. From the time-dependent force and cantilever height information, the amplitudes of effective tension, surface area strain, phase shift and an complex elastic modulus are derived (Fischer-Friedrich et al., 2016).

A widely used method to assess cortex tension is **micropipette aspiration** (Figure 1.5). Thereby a micropipette is brought into contact with individual cells and a suction pressure is applied that draws the cell partly into the pipette as observed by light microscopy (Evans and Yeung, 1989; Evans and Robinson, 2018). The pressure is stepwise increased until the aspirated length of the cell pulled into the pipette equals the pipette radius and resembles a hemi-circle. At that (critical) pressure, the surface tension can be calculated applying Laplace law (Larson et al., 2010; Chaigne et al., 2013, 2015). Rising the suction pressure beyond that critical value results in a liquid-like flowing of the cell into the pipette and allows for characterization of viscoelastic properties of the cell (Hochmuth, 2000; Reichl et al., 2008). Micropipettes can also be used to manipulate the cortex of mitotic cells (Sedzinski et al., 2011).

A contact-free interrogation of the cell’s mechanical properties is enabled by the **Optical Stretcher** (Figure 1.6; Guck et al., 2001, 2005). This technique uses a dual beam laser trap to trap and deform cells within a microfluidic channel. By increasing of the laser power above trapping power, stress is induced on the cell surface due to momentum transfer, which results in cell deformation along the laser axis, quantified by the axial strain. Compliance can be calculated by dividing the strain by the calculated optical stress, taking also a geometrical factor into account (Ekpenyong et al., 2012; Matthews et al., 2012). Also, viscoelastic properties can be studied by fitting time dependent creep compliance curves to a mechanical models (Guck et al., 2001; Lincoln et al., 2007). While optical stretcher enables trapping and manipulation of entire cells, **optical tweezers** present highly sensitive tools for probing forces within subcellular compartments (Ashkin et al., 1987; Charlebois et al., 2011; Ferraro-Gideon et al., 2013) although their force range is rather limited.

High throughput mechanical probing of suspended cells in a contact-free manner can be facilitated by real-time **deformability cytometry** (RT-DC) (Figure 1.7). Using RT-DC, rates of higher than 100 cells/sec can be reached (Otto et al., 2015). Suspended cells are passed through a microfluidic channel, where they are hydrodynamically deformed. Several parameters including deformation and area are analyzed on the fly. Post-processing using analytical or numerical models are employed to calculate for instance an apparent elastic modulus (Mietke et al., 2015; Mokbel et al., 2017; Rosendahl et al., 2018; Matthews et al., 2020). Another type of microfluidic assay makes use of a hollow microchannel resonator, oscillating at its resonance frequency, through which cells are passed. Depending on the position within the channel, changes in the resonance frequency could be attributed to acoustic scattering correlating with changes in mechanical properties of mitotic cells (Kang et al., 2019).

Non-invasive techniques that are also suitable to study mechanical properties of cells within a tissue or organoid are **acoustic microscopy** and **Brillouin microscopy**. In **acoustic microscopy** (Figure 1.9) ultra high-frequency sound waves (MHz-GHz range) passing through cells and tissues are analyzed and parameters such as sound velocity and attenuation and sample thickness are measured. The obtained sound velocity can be related to the compression modulus and thereby reflects on the cell mechanical properties (Kundu et al., 2000; Bereiter-Hahn, 2005). **Brillouin microscopy** (Figure 1.8) relies on the effect of Brillouin scattering, which occurs due to the interaction of incident laser light with acoustic phonons, causing a frequency shift that can be related to the longitudinal modulus and therefore sample compressibility (Scarcelli and Yun, 2008, 2012; Prevedel et al., 2019). Both techniques have already been applied for the study of mitotic cells (Pasternak et al., 2015; Liu et al., 2019).

The forces exerted during cell rounding can be quantified using atomic force microscopy (AFM). Here a flat cantilever is positioned above a cell in prophase. As the cell rounds and comes into contact with the cantilever, the forces exerted onto the cantilever can be read out (Figure 1.3 and Box 1; Stewart et al., 2011a). Using this method, changes in rounding forces were monitored over the course of mitosis, revealing an increase during progression from prometaphase to metaphase, before, at cytokinesis, the cells loose contact with the cantilever when elongating and flattening again (Stewart et al., 2011a).

Follow-up studies that combined AFM with confocal microscopy enabled the measurement of surface tension and intracellular pressure changes in mitotic cells that had been pre-rounded by detachment from the substrate (Fischer-Friedrich et al., 2014; Cattin et al., 2015; Ramanathan et al., 2015). Surface tension and intracellular pressure were calculated by applying Laplace’s law (see Glossary) which was found to be well suited to describe the observed shapes under uniaxial compression (Fischer-Friedrich et al., 2014; Ramanathan et al., 2015). Since the contribution of membrane tension is considered to be negligible (Clark et al., 2013; Chalut et al., 2014; Chugh and Paluch, 2018), the observed changes in surface tension are mainly due to changes in cortical tension arising from the cortical acto-myosin network (see Glossary) (reviewed in Kelkar et al., 2020). Uniaxial AFM confinement (Figure 1.4) of rounded, non-adherent cells showed that cortical tension is greatly increased in mitosis compared to interphase (Fischer-Friedrich et al., 2014; Ramanathan et al., 2015; Chugh et al., 2017). In HeLa cells, it was found to increase from 0.2 mNm^–1^ during interphase to 1.6 mNm^–1^ in metaphase (Fischer-Friedrich et al., 2014). Crucially, as this technique involves lowering an AFM cantilever onto rounded, non-adherent cells either in interphase or arrested in mitosis, these studies demonstrate that the cortical tension increase observed at mitosis happens independently of cell shape changes. These studies also revealed that entry into mitosis is associated with an ∼10-fold increase in intracellular pressure (Fischer-Friedrich et al., 2014; Ramanathan et al., 2015). A more in-depth characterization of the mechanics of the mitotic cortex by stepwise uniaxial compression under an AFM cantilever (Figure 1.4) confirmed that the deformation response measured is dominated by the cortical layer (and not the bulk of the cytoplasm) so that it fits best a view of the cell as a visco-elastic shell surrounding an incompressible bulk liquid cytoplasm (Fischer-Friedrich et al., 2016).

In the simplest case, cortical tension and intracellular pressure should be directly coupled (according to Laplace’s law – see Glossary). As a result, an increase in cortical contraction will raise intracellular pressure causing water efflux. This in turn will cause an increase in the osmotic pressure difference between the inside and outside. Nevertheless, because the osmotic forces involved are orders of magnitude higher than those generated by cortex contractility, the volume changes involved are expected to be negligible (Clark et al., 2014; Fischer-Friedrich et al., 2014). This is supported by data from experiments in which induced changes in acto-myosin contractility did not change cell volume (Stewart et al., 2011a; Zlotek-Zlotkiewicz et al., 2015; Cadart et al., 2018).

Cells do undergo changes in volume though as they pass into and out of mitosis. This they achieve by actively pumping ions across the plasma membrane. Thus, when cell volume was accurately measured during mitotic rounding using fluorescence exclusion microscopy, cell volumes were found to increase by 10–30% over a period of several minutes following the transition from prophase to prometaphase (Zlotek-Zlotkiewicz et al., 2015; Cadart et al., 2018). A similar increase in cell volume that depended on Na^+^H^+^ ion exchange was seen when single cells were weighed in medium using a suspended microchannel resonator (Son et al., 2015). Along with nuclear permeabilization, this entry of water into the cell at mitotic entry may also contribute to the dilution of cytoplasmic proteins (Mchedlishvili et al., 2018). As a result, the mitotic cytoplasm is likely to be less viscous and more homogeneous than in interphase, a process that is also aided by fragmentation of subcellular compartments such as mitochondria and endoplasmic reticulum (Champion et al., 2017). This may be important for chromosome segregation (Moulding et al., 2012). Osmotic pressure changes due to ion fluxes across the membrane in the mitotic cell and concomitant water influx have been suggested to drive intracellular pressure increase in early mitosis to aid cell rounding, while the acto-myosin cortex actively guides shape changes and controls surface tension (Stewart et al., 2011b).

## Mitotic Cells Are Characterized by a Stiffer Cortex

The changes in cortical tension, the viscoelastic properties of the cortex and its geometry all affect the response of the cell to deformation as revealed by probing the cell surface locally using an indenter. Most studies use AFM, although simple measurements such as cortical stiffness can also be obtained by pushing calibrated microneedles (Figure 1.1 and Box 1) onto the cell surface and measuring deflection of the needle tip (Maddox and Burridge, 2003). The resulting AFM force-indentation curves are then typically fitted with the Hertz/Sneddon model (Hertz, 1882; Sneddon, 1965) to derive an apparent Young’s modulus (see Glossary, Figure 1.2 and Box 1), thereby treating the cell as a homogenous (visco)elastic material. Since the indentation depth reached is typically less than 500 nm, the response to deformation is dominated by the cell cortex, so the apparent Young’s modulus (see Glossary) is usually taken as an indirect measure of cortical stiffness (Radmacher et al., 1996; Matzke et al., 2001; Salbreux et al., 2012). The measured cortical stiffness includes but does not differentiate well the relative contributions of cortex viscoelasticity and cortex tension, and is further influenced by cortical thickness. However, in most of the studies, changes in cortical stiffness correlate well with cortical tension changes, because of the short time-scale of the deformation. In this way, local surface mapping of fly cells using AFM (Figure 1.2) revealed a ∼4-fold increase in apparent Young’s Modulus when cells entered mitosis (Kunda et al., 2008), with further stiffening observed at the furrow when cells divided (Matzke et al., 2001; Kunda et al., 2008). Similarly, when analyzing human breast epithelial cells using AFM, a 2–4-fold increase in apparent elastic modulus between interphase and mitosis was observed that was independent of cell shape (Matthews et al., 2020; Figure 1.2).

This increase in cortical stiffness at the local level also translates to a resistance to whole-cell deformation. Mitotic cells were also found to be less compliant and therefore stiffer than interphase cells using an Optical Stretcher (Matthews et al., 2012; Figure 1.6 and Box 1). Similarly, using real-time deformability cytometry (RT-DC), a high-throughput microfluidic technique to assess cell mechanics (Figure 1.7), mitotic MCF10A cells were found to be less deformable than their interphase counterparts (Matthews et al., 2020). In fact, the difference between the interphase and mitotic population was so pronounced that it allowed for label-free high-throughput mechanical phenotyping (Rosendahl et al., 2018). These studies all point to a clear difference in the biophysical properties of cells between mitosis and interphase that is independent of cell shape and adhesion: cells in mitosis are stiffer and more able to resist deformation.

## Changes in Cortical Actin and Myosin Architecture Underpin Changes in Mechanics

How are these forces and the changes in cortical mechanics generated? This depends on the acto-myosin cortex, a network of filaments and contractile elements that is coupled to the plasma membrane (Chugh and Paluch, 2018; Kelkar et al., 2020). Depolymerization of actin filaments completely removes any mechanical difference between interphase and mitotic cells, resulting in cells that are extremely compliant (Stewart et al., 2011a; Matthews et al., 2012; Ramanathan et al., 2015; Fischer-Friedrich et al., 2016; Chugh et al., 2017) while the disassembly of the mitotic spindle has little effect on cortical mechanics (Stewart et al., 2011a; Fischer-Friedrich et al., 2016). The acto-myosin network plays a fundamental role in controlling cell shape, mechanics and the ability to exert force and resist deformation in mitosis. But how is this orchestrated at a molecular level?

**FIGURE 2:**
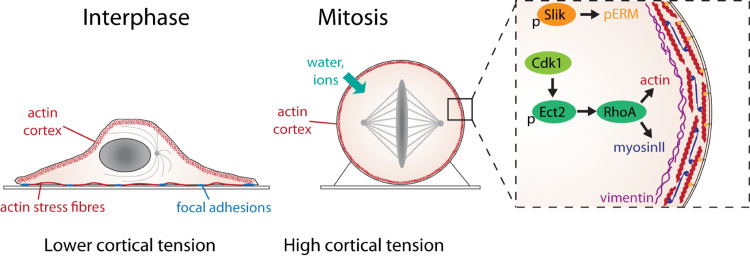
The molecular basis of mitotic force generation. The transition from interphase **(left)** to mitosis **(center)** in a single adherent cell in tissue culture is accompanied by loss of substrate adhesion, an increase in acto-myosin cortical tension and an increase in intracellular pressure due to water influx. The box shows the molecular changes that control cortical tension. Activation of Ect2 by Cdk1 phosphorylation and nuclear export leads to the activation of RhoA, which leads to the assembly of actin filaments (red) and myosin II mini-filaments (blue) at the cell cortex. The rigid, contractile acto-myosin cortex is attached to the plasma membrane by ERM proteins (orange), activated in mitosis through phosphorylation by the kinase, Slik. A network of intermediate filament protein, vimentin (purple), underlies the cortical actin network, which also contributes to cortical tension.

Recent studies have demonstrated that differences in cortical architecture, contractility and protein composition likely contribute to the differences in biophysical properties between interphase and mitotic cells (summarized in Figure 2). In mitosis, the cortex is thinner than in interphase, despite having higher tension (Chugh et al., 2017). By perturbing actin regulators, Chugh et al. (2017) suggested that a key factor determining cortical tension was actin filament length. Treatments that shortened filaments, such as knockdown of nucleator, Diaph1, led to a thinner cortex (Chugh et al., 2017) and decreased cortical tension in mitosis (Rosa et al., 2015; Chugh et al., 2017; Toyoda et al., 2017). Conversely, lengthening filaments by removing actin capping protein, CAPZB, or filament severer, cofilin, led to a thicker cortex but also decreased tension (Chugh et al., 2017). This suggests that the mechanical changes observed in mitosis may in part be due to changes in filament length, achieved by altering the balance between filament assembly, disassembly, branching, severing and capping. Filament nucleation in the cortex depends on the coordinated activity of Arp2/3, which forms branched filaments, and Diaph1, which nucleates linear filaments (Bovellan et al., 2014; Cao et al., 2020). Proteomics studies have shown that the cortex contains numerous regulators of filament assembly, severing, capping and bundling (Biro et al., 2013; Bovellan et al., 2014; Serres et al., 2020). High through-put mechanical screens have found that many of these are required to generate cortical tension in mitosis (Toyoda et al., 2017; Rosendahl et al., 2018). In addition, crosslinks are critical. A computational simulation of the cortex structure predicts that connectivity is key to achieving high tension across a network in a way that depends on filament lengths, turnover rates and crosslinks (Chugh et al., 2017). Network turnover and filament cross-linking modulate network connectivity (Koenderink and Paluch, 2018). Thus, the depletion of actin filament cross-linkers, including fascin and alpha-actinin, decrease mitotic cortical tension (Fischer-Friedrich et al., 2016; Toyoda et al., 2017). Cortical recruitment, activation or de-activation of specific proteins in mitosis can also profoundly alter the mechanical properties of the network as a whole. For example, WDR1, a protein that promotes actin filament disassembly is enriched within the mitotic cortex (Serres et al., 2020) and is required for mitotic rounding (Fujibuchi et al., 2005; Matthews et al., 2012).

## Cortical Myosin

One of the major functions of actin filaments in the cortex is to support myosin contractility. Myosin mini-filaments within the network provide crosslinks but also act as motors to slide filaments over each other. Myosin II accumulates progressively at the cortex during mitotic rounding corresponding to an increase in rounding pressure (Ramanathan et al., 2015). This accumulation is essential for generating cortical tension as myosin II depletion reduces the ability of cells to apply pressure in mitosis by 90% (Toyoda et al., 2017). However, treatment of cells with blebbistatin, which inhibits myosin motor activity but does not affect its accumulation at the mitotic cortex (Ramanathan et al., 2015) or cortex thickness or turnover rate (Ramanathan et al., 2015; Chugh et al., 2017) has a more subtle effect. In rounded cells confined under an AFM cantilever (Figures 1.3,1.4), blebbistatin-treated cells were initially able to resist the applied stress but not to sustain the pressure (Ramanathan et al., 2015). Detailed rheological characterization revealed a more solid-like but less stiff cortex with blebbistatin treatment (Fischer-Friedrich et al., 2016). On the other hand, increasing contractility using RhoA activator Calpeptin raises intracellular pressure and cortical tension and induces blebbing in mitosis (Ramanathan et al., 2015). Blebs, which often form under conditions of high contractility or cortical instability (Tinevez et al., 2009), are rarely seen in early mitosis, suggesting that intermediate levels of contractility are optimal for maximum cortical tension. It seems likely that myosin is required to increase cortical tension in mitosis through its dual roles in promoting network crosslinking and in applying contractile forces to the network. In reality, these two functions cannot be easily separated as network connectivity also affects contractility (Ennomani et al., 2016; Koenderink and Paluch, 2018).

## Cortex/Membrane Cross-Linking

The mitotic cortex is also crosslinked to the plasma membrane. By tethering these two structures, these crosslinks help to generate the mechanically stiff cortex. While several proteins have the ability to link the membrane and the actin cytoskeleton in different contexts (e.g. spectrin in red blood cells), the ezrin/radixin/moesin (ERM) family of proteins is likely to be critical in mitosis. Members of this family are widely expressed at high levels, bind both the actin cortex and transmembrane proteins within the plasma membrane, and are activated by phosphorylation and specifically recruited to the cell cortex in mitosis in both fly and human cells (Carreno et al., 2008; Kunda et al., 2008; Roubinet et al., 2011; Machicoane et al., 2014). In fly cells, the mitotic activation of moesin (the single fly ERM homolog) is essential for mitotic stiffening (Kunda et al., 2008). While the picture in mammalian cells is less clear, partly because of redundancy, ezrin silencing leads to a slight decrease in cortical tension (Toyoda et al., 2017). ERM proteins are likely to play an important conserved role in the regulation of cortical mechanics, as there is additional evidence that they regulate membrane-cortical interactions to maintain mammalian cell shape (Clark et al., 2014), regulate bleb formation and contraction during mitosis (Tinevez et al., 2009; Sedzinski et al., 2011) and become polarized at mitotic exit to aid cell division (Roubinet et al., 2011; Kunda et al., 2012).

## The Role of Vimentin

The acto-myosin cortex is key to generating mitotic cortical tension. However, two recent studies revealed the involvement of a second network of the intermediate filament protein, vimentin, which lies just beneath the actin cortex in mitotic cells. Serres and colleagues compared the proteins bound to actin in interphase and mitosis using mass spectroscopy and found that vimentin was specifically enriched in mitosis in HeLa cells (Serres et al., 2020). Duarte et al. (2019) showed that vimentin is recruited to the acto-myosin cortex via its c-terminal tail region and is required for normal mitotic progression. The presence of a vimentin layer underlying the actin network appears to both organize and strengthen the acto-myosin cortex, since vimentin knockdown led to the formation of a thicker, more disorganized cortex with lower tension (Serres et al., 2020). While this new role of vimentin in modulating the mechanical properties of the acto-myosin cortex during mitosis is interesting, many cell types, including epithelial cells, do not express vimentin. Furthermore, the over-expression of vimentin in MCF-7 cells, where it is not normally expressed, was not sufficient to drive its cortical localization (Duarte et al., 2019), hinting at different regulatory mechanisms between different cell types.

## What Triggers Cortical Changes at Mitosis?

The mechanical changes associated with mitosis are striking in that they occur over a rapid timescale (10 min) and reverse just as quickly at mitotic exit (Stewart et al., 2011a). Increasing cortical tension can be viewed as part of a suite of changes that occur at mitotic entry and affect almost all cell structures including DNA, internal membranes and organelles. All are driven by the phosphorylation of multiple proteins by mitotic kinases, including the master regulator Cdk1/Cyclin B. Cdk1 activity rapidly ramps up during prophase at the same time as mitotic rounding (Gavet and Pines, 2010). Mitotic stiffening is dependent on Cdk1, as the addition of a Cdk1 inhibitor results in a rapid decrease of intracellular pressure and cortical tension as cells exit mitosis (Ramanathan et al., 2015). Cdk1 and other mitotic kinases phosphorylate a huge number of substrates (Blethrow et al., 2008) including many that have the potential to control cell shape and mechanics. Cdk1 drives the disassembly of focal adhesion complexes and associated non-cortical actin structures, such as stress fibers, required for mitotic rounding (Dao et al., 2009; Jones et al., 2018; Lock et al., 2018). Some cortical regulators are directly phosphorylated by Cdk1 including vimentin (Yamaguchi et al., 2005) and WDR1 (Fujibuchi et al., 2005). However, many of the changes to acto-myosin organization are thought to be driven by the activation of RhoA in mitosis (Maddox and Burridge, 2003). Ect2, a RhoGEF, plays a key role in regulating the changes in mitotic cell shape and mechanics that accompany mitotic progression. In interphase, Ect2 resides in the nucleus but as cells enter mitosis, it is exported into the cytoplasm following phosphorylation by Cdk1, where it activates RhoA at the plasma membrane to trigger mitotic rounding (Matthews et al., 2012). AFM measurements have demonstrated that both Ect2 and RhoA are required for cortical stiffening (Maddox and Burridge, 2003; Matthews et al., 2012) and rounding force generation (Ramanathan et al., 2015). Ect2 knockdown blocks the accumulation of both actin and myosin at the cell cortex in mitosis (Matthews et al., 2012; Toyoda et al., 2017), so is likely to be required for both filament organization and contractility during mitotic rounding. The Ect2-RhoA pathway controls mitotic rounding and stiffening by multiple mechanisms including driving contractility at the cell edge during mitotic rounding (Ect2-depleted cells round up more slowly (Matthews et al., 2012), promoting actin filament nucleation through the activation of the formin Diaph1 (Rosa et al., 2015) and organizing cortical filaments. In a parallel pathway, ERM proteins are activated by phosphorylation by the kinase Slik to link the cortical actin network to the plasma membrane, ensuring its stability and rigidity (Carreno et al., 2008; Kunda et al., 2008; Machicoane et al., 2014).

Once cells reach anaphase, cyclin B degradation and the resulting decline in Cdk1 activity coincide with the reverse process as rounding pressure drops (Stewart et al., 2011a). In preparation for cell division, the previously uniform cortex becomes polarized, so that the furrow stiffens (Matzke et al., 2001; Kunda et al., 2008) while the poles soften and relax – allowing cells to elongate. This symmetry-breaking event is driven by a rapid change in the localization of proteins controlling cortical mechanics, which lose their uniform distribution at anaphase. As cells exit from mitosis, Ect2 becomes localized to the spindle midzone where it activates RhoA to assemble the acto-myosin contractile ring (Somers and Saint, 2003; Yüce et al., 2005; Su et al., 2011). At the same time, the chromosomes move poleward carrying the kinetochore-localized phosphatase PP1/sds22 (Rodrigues et al., 2015) which dephosphorylates polar ERM proteins and RanGTP (Kiyomitsu and Cheeseman, 2013) allowing clearance of cortical actin and polar relaxation (Roubinet et al., 2011; Kunda et al., 2012). Pressure is released by membrane blebbing at the poles, which help to stabilize cell shape during division (Sedzinski et al., 2011). In summary, the structural changes to the mitotic spindle induce a symmetry breaking event in the uniform mitotic cortex to polarize the cell to divide in two. Actin re-arrangements at mitotic exit have been comprehensively reviewed elsewhere (Green et al., 2012; Ramkumar and Baum, 2016).

While many of the molecular mechanisms driving mitotic actin rearrangements are known, it is not so clear what regulates the water influx to increase intracellular pressure at mitosis. An RNAi screen that probed cell mechanics using a wedged AFM cantilever (Figure 1.4) identified several ion channels that when knocked down decreased cell rounding force (Toyoda et al., 2017). This study also found an enzyme, DJ-1, a glycoxylase involved in mitochondrial regulation and stress response, required for osmotic pressure maintenance in mitosis (Toyoda et al., 2017). There are reports that the Na^+^H^+^ antiporter required for mitotic swelling may be regulated by RhoA (Hooley et al., 1996). However, much work needs be done to identify the mechanisms that control mitotic swelling and, given swelling alone is not sufficient for force generation, to determine how these changes are coordinated with cortical actin rearrangements. Given the role of cortical tension in counteracting and balancing intracellular pressure, it may be that the two pathways are interlinked and thus hard to separate.

## The Function of Mitotic Stiffening: Generating Space to Divide

The mechanical changes in early mitosis allow cells to exert force on their environment. In single adherent cells, this can be measured as force on an AFM cantilever (Figure 1.4; Stewart et al., 2011a; Cattin et al., 2015; Ramanathan et al., 2015). In a non-polarized 3-dimensional (3D) environment, however, such as a hydrogel, forces will act isotropically (Nam and Chaudhuri, 2018). Within a tissue, the directionality of the force depends on tissue architecture. In a flat, stretched epithelium rounding cells may pull on their neighbors, while in a dense crowded tissue, they are more likely to have to push. Pulling and pushing forces associated with mitotic rounding have been quantified using traction force microscopy in epithelial monolayers (Uroz et al., 2018) and by the deformation of micro-fabricated pillars surrounding rounding cells (Sorce et al., 2015). Because of their increased cortical stiffness, mitotic cells are more resistant to cell deformation and therefore tissue-level forces. Indeed, in an epithelial monolayer subjected to a constant stretch, interphase cells became elongated while mitotic cells remained spherical (Wyatt et al., 2015). But does the ability of mitotic cells to apply or resist force serve a purpose during cell division? And do the forces associated with mitotic rounding have a function *in vivo* where cells divide surrounded by other cells and extracellular matrix (ECM)?

## Lessons From Confinement Studies

The function of mitotic rounding and stiffening is not immediately apparent for cells dividing in tissue culture conditions where space is unlimited, rather it is revealed under crowded conditions. Various studies have used mechanical and geometric constraints to limit the ability of cells to round up at mitotic entry, including confining cells under AFM cantilevers (Cattin et al., 2015), under a low (<5 μm) roof (Tse et al., 2012; Lancaster et al., 2013) or in thin microchannels (Xi et al., 2016; Cadart et al., 2018). In each case, confinement generates multiple defects in mitosis. In flattened cells, the mitotic spindle is unable to efficiently capture chromosomes due to an upper limit in microtubule reach: the mitotic spindle cannot rescale to account for the altered geometry and is unable to contact all chromosomes (Dumont and Mitchison, 2009; Lancaster et al., 2013). Since satisfaction of the spindle assembly checkpoint requires attachment of every chromosome (Musacchio, 2015), this leads to prolonged mitotic arrest (Lancaster et al., 2013; Cattin et al., 2015). Cells confined in a low chamber also have difficulty resolving their mitotic spindle to form a bipolar structure, which frequently leads to pole splitting and cell division into three or more daughter cells rather than two (Tse et al., 2012; Lancaster et al., 2013). These catastrophic errors in spindle formation reveal a role for mitotic rounding in generating space in which to assemble a proper mitotic spindle, itself a bulky 3D structure (Cadart et al., 2014). There appears to be a critical threshold of cell height (5–10 μm, depending on cell type, size & genome content) below which a proper mitotic spindle is unable to form (Lancaster et al., 2013; Cadart et al., 2014; Cattin et al., 2015). Mitotic cell rounding not only affects DNA segregation but also the accurate partitioning of cytoplasmic contents between two daughter cells. In cells confined in thin, narrow microchannels, the mitotic spindle is unable to properly position in the cell center resulting in asymmetrical cell division (Xi et al., 2016; Cadart et al., 2018).

Confining cells under a rigid surface demonstrates the function of mitotic rounding in creating a spherical space for spindle assembly. However, the function of mitotic stiffening and force generation becomes clear when cells are confined under a deformable material (Figure 1.10). Cells confined under soft polyacrylamide gels (∼ 5 kPa) are able to generate forces to deform the gel and create the space needed for spindle assembly (Lancaster et al., 2013). However, under a stiff gel that resists mitotic rounding (∼ 30 kPa), multiple errors ensue, similar to those observed in limited height chambers (Lancaster et al., 2013). Treatments that abolish the ability of the cell to generate contractile forces such as actin depolymerization or ROCK inhibition lead to chromosome segregation errors even under softer gels (Lancaster et al., 2013; Matthews et al., 2020). Similarly, in 3D, cells embedded in viscoelastic alginate hydrogels generate forces in all directions as they round up, and stiff viscoelastic gels characterized by slow stress relaxation disrupt cytokinesis by preventing cell elongation (Nam and Chaudhuri, 2018). Thus, the ability of mitotic cells to apply force is crucial in stiff and confining environments to generate the space needed to divide.

## The Mechanics of Dividing in a Tissue

**FIGURE 3:**
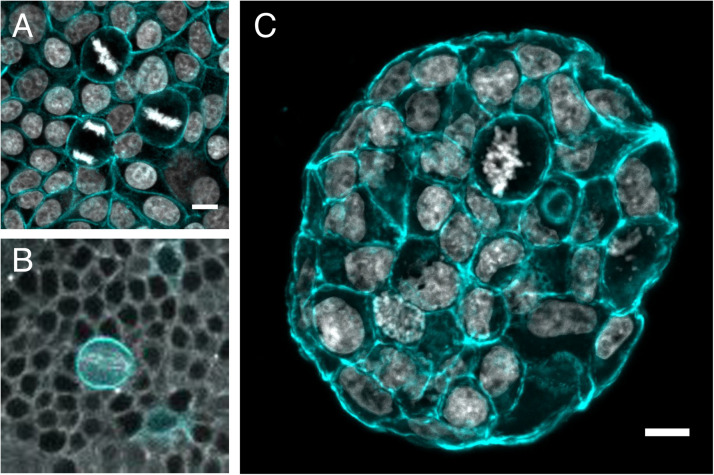
Mitotic rounding in tissues and tumoroids. Examples of mitotic cells rounding while surrounded by other cells **(A)** in a non-transformed confluent epithelial cell monolayer (MCF10A) plated on a soft polyacrylamide hydrogel, stained with phalloidin-TRITC to visualize actin (cyan) and DAPI to visualize DNA (Gray) (Image by HM), **(B)** *in vivo* in a mitotic sensory organ precursor cell (labeled with LifeAct-GFP in cyan) in the notum of the developing *Drosophila* pupa. The whole tissue is labeled with tubulin (gray) to stain the mitotic spindle. (Image by Nelio Rodrigues) and **(C)** frozen section of an MCF-7 tumor spheroid grown for 14 days within a PEG/heparin hydrogel in 3D, stained with phalloidin-TRITC (cyan)/DAPI(gray) for F-actin/nuclei (image by AT). Scale bars are 10 μm.

Rounded mitotic cells, which deform their interphase neighbors are commonly observed *in vivo* during development and in many different adult tissues (Luxenburg et al., 2011; Nakajima et al., 2013; Hoijman et al., 2015; Rosa et al., 2015; Freddo et al., 2016; Chanet et al., 2017; Aguilar-Aragon et al., 2020 see Figure 3 for examples). However, the mechanics of mitosis has not been well studied in the tissue context. Cell division within a multicellular organism adds an extra layer of complexity: in culture, cells detach from the substrate to round up (Dao et al., 2009; Dix et al., 2018), but *in vivo*, cells must maintain attachment to their neighbors to ensure tissue integrity. How exactly cells create space to divide while maintaining tight attachment to their neighbors varies depending on tissue type, density and organization. The majority of studies on mitotic rounding in developing and adult proliferative tissues have focused on epithelial tissues of different types. In simple epithelial monolayers on glass, cells round up in mitosis, often appearing to deform neighboring cells (Figure 3A) but adherens junctions persist throughout to maintain epithelial integrity (Baker and Garrod, 1993; Reinsch and Karsenti, 1994; Gloerich et al., 2017; Hart et al., 2017). Similarly, *in vivo*, in simple cuboidal epithelia in the developing fly (Figure 3B), mitotic cells use the forces generated by acto-myosin to round up to the apical surface of the epithelium (Rosa et al., 2015), and retain most of their apical cell-cell junctions throughout mitosis (Aguilar-Aragon et al., 2020). As in cell culture, these changes depend on release of Ect2 from the nucleus at mitotic entry (Rosa et al., 2015) and the cortical activation of the fly ERM protein moesin (Kunda et al., 2012).

Cells in other epithelial tissue types face different challenges in rounding up to divide. Tall, thin cells in pseudo-stratified epithelia undergo a process known as interkinetic nuclear migration prior to mitosis where the nucleus and cell body migrate to the apical surface (Meyer et al., 2011; Norden, 2017) while maintaining attachment to the basal lamina via a thin basal process (Kosodo et al., 2008). In the pseudostratified epithelium of the fly wing disc, this process depends on accumulation of actin and activated moesin at the cell cortex (Nakajima et al., 2013) as well as local tissue tension (Kirkland et al., 2019). However, in this system, unlike in cuboidal epithelia, adherens junctions are disassembled during mitosis (Aguilar-Aragon et al., 2020). Interestingly, in this case the loss of adherens junctions is co-ordinated by the same pathways that control mitotic rounding, including activation of Ect2 and RhoA at mitotic entry (Aguilar-Aragon et al., 2020). This fits with observations that RhoA and Ect2 can themselves control the stability and turnover of cell junctions via endocytosis (Levayer et al., 2011; Ratheesh et al., 2012). In the case of fly pseudo-stratified epithelia, tissue integrity can be maintained even in the absence of adherens junctions due to the persistence of septate junctions through mitosis (Nakajima et al., 2013). These studies reveal that many of the key molecular players, including actin, myosin, Ect2, and ERM proteins, that control mitotic shape in single cells also control mitotic rounding in tissue, although there are clearly differences in their regulation that depend on cell type and tissue architecture.

While mitotic rounding has been studied *in vivo*, there have been few attempts to measure the associated forces. An AFM-based, parallel plate approach has been used to measure the forces applied by neuroblasts, isolated from fly brains, when they entered mitosis, demonstrating that primary cells exert rounding pressure (Pham et al., 2019). Alternatively, monolayers of epithelial cell lines have been used to model tissue. In MDCK monolayers on a soft substrate, traction force microscopy shows that mitotic cells exert forces both on neighboring cells and on the substrate as they round up and divide (Uroz et al., 2018). Sorce and colleagues used the deformation of soft micropillars to measure the outwards force applied by MDCK cells as they entered mitosis. As with single cells, they found that cells pushed outwards in mitosis with a force that peaked at metaphase and required acto-myosin contractility and osmotic pressure (Sorce et al., 2015). This allowed cells to displace or escape the pillars, which otherwise act as physical obstacles causing defects in spindle formation and chromosome segregation (Sorce et al., 2015). A computational model of this type of scenario in which cells round within a crowded epithelial monolayer suggests that the expansion of mitotic cells could reflect the difference in hydrostatic pressure between mitotic cells and their interphase neighbors (Nematbakhsh et al., 2017).

Measurement of the mechanical alterations of the cortex during mitosis and their impact on spindle formation and mitotic progression in a tissue context remain to be addressed. This is, in part, because most assays used to determine cell mechanical properties require cells to be in suspension (micropipette, AFM, microfluidics), or use direct contact with the cell surface (Cantilever, pipette or bead) (see Figure 1). Thus, measuring mitotic mechanics *in vivo* will require the appropriation and development of new contact-free measurement techniques. For instance, acoustic microscopy has been employed to map mechanical properties of cells and tissues (Kundu et al., 2000; Bereiter-Hahn, 2005; Figure 1.9). Pasternak et al. employed acoustic microscopy to study MCF-7 cells through the cell cycle (Pasternak et al., 2015). A drop in the mechanical properties (quantified as adiabatic bulk modulus) from G1/G2 to metaphase was detected, but this could not be directly related to specific cellular components and requires further characterization. Another new technique that is being used to measure the mechanical properties of cells and tissues is confocal Brillouin microscopy (Scarcelli and Yun, 2008, 2012; Box 1). In those cases, the sample is scanned by a laser beam and a frequency shift due to Brillouin scattering is detected which is related to the mechanical properties of the sample (Scarcelli and Yun, 2008, 2012). Thus far, in the context of mitosis, Brillouin microscopy was applied to single cells where differences in the Brillouin shift between interphase and mitosis were observed (Liu et al., 2019; Figure 1.8), but the technique has not yet been used to study mitotic cells within complex tissues.

## Mitotic Rounding in Tissue Homeostasis and Development

It is becoming increasingly clear that the mitotic shape changes in a tissue context often serve a wider purpose, beyond space creation for spindle assembly, by contributing to morphogenesis. For example, the shape of dividing cells often plays a role in polarizing the division axis. In a tissue, the position of the mitotic spindle determines the division axis, which is crucial for cell packing. Cell divisions within the plane of the epithelium maintain tissue integrity and homeostasis, while perpendicular divisions can drive the formation of new layers and cell differentiation (Williams and Fuchs, 2013; Ragkousi and Gibson, 2014). Disruption of acto-myosin in mitosis to prevent mitotic rounding leads to mis-orientation of the spindle and therefore the cell division axis in fly epithelia (Chanet et al., 2017; Lam et al., 2020) and in the mouse epidermis (Luxenburg et al., 2011). In the developing fly wing disc, acto-myosin-dependent mitotic rounding is essential to keep division within the epithelial plane to prevent cell delamination and apoptosis (Nakajima et al., 2013). Similarly, in the mouse epidermis, reduced acto-myosin contractility leads to impaired spindle orientation and biases divisions toward the perpendicular axis, forcing ectopic differentiation (Dias Gomes et al., 2019), Interestingly, in this study disruption of mitotic rounding also led to defects in spindle formation including lagging chromosomes and tripolar divisions (Dias Gomes et al., 2019), similar to those seen in confined single cells (Lancaster et al., 2013). In addition to parallel/perpendicular division orientation, the angle of division within the epithelial plane can also affect tissue morphology, for example by promoting uniaxial growth and elongation or to relieve tissue tension (Wyatt et al., 2015). Spindle orientation in symmetrically dividing cells relies on a combination of sensing of cortical cues, cell junctions and cell shape as well as mechanical factors and has been comprehensively reviewed elsewhere (di Pietro et al., 2016; van Leen et al., 2020). In addition, changes in cell shape and mechanics during division play a role in asymmetrical cell division, where unequal segregation of cytoplasmic or cortical factors results in two daughter cells of different fate and also, frequently, of different size. The size asymmetry in dividing *Drosophila* neural stem cells was found to be generated by an unequal re-distribution of force-generating molecules at anaphase: the larger daughter cell had increased hydrostatic pressure, while the smaller had increased myosin localization and contractility (Roubinet et al., 2017; Pham et al., 2019). In Zebrafish neural progenitors, daughter cell fates depended on the asymmetrical interphase localization of membrane ligands being maintained during mitotic rounding (Akanuma et al., 2016).

Finally, there have been multiple examples of mitotic rounding contributing to morphogenesis during embryonic development. The forces exerted by cells as they round up in mitosis are able to deform epithelial sheets to produce new structures, for example, to drive the invagination required to produce tubular structures in the developing fly trachea (Kondo and Hayashi, 2013) or for lumen formation during Zebrafish inner ear development (Hoijman et al., 2015). In the developing mouse epithelium, an invagination produced by a cell rounding up at mitosis patterns the tissue by marking the position where a villus will form (Freddo et al., 2016). During gastrulation in the early chick embryo, cortical acto-myosin during division can drive the cell intercalations required for tissue scale movement (Firmino et al., 2016). In early Zebrafish morphogenesis, cell-cell contact weakening during mitotic rounding has the effect of fluidising the tissue, which is essential for blastodermal spreading (Petridou et al., 2019). These studies reveal that mitotic rounding plays diverse roles in both maintaining force balance and integrity in homeostatic tissue and in driving morphogenetic movements during development. However, the relative contribution of cell mechanics has not been addressed, due to the difficulty of making mechanical measurements *in vivo*. It is likely that the mechanical differences between mitotic cells and their interphase neighbors are an important factor in many of these processes and one that will be the subject of future work.

## Mitotic Rounding and Stiffening in Diseased Tissue

The ability of cells to apply force on their surroundings and maintain a stable round shape during mitosis is likely to be more important, the stiffer the extracellular environment. Indeed, cells dividing in extremely stiff, elastic 3D gels suffer multiple division defects (Nam and Chaudhuri, 2018). Many disease states result in changes to tissue mechanics. Tissue damage-induced fibrosis in many inflammatory conditions results in tissue stiffening and is also a risk factor for the development of cancer (Boyd et al., 1998; Paszek et al., 2005). Although, at the individual cell level, invasive cancer cells are typically less stiff than their non-transformed counterparts (Guck et al., 2005; Rother et al., 2014; Lekka, 2016; Alibert et al., 2017), tumors are normally far stiffer than healthy tissue due to cell over-proliferation, extracellular matrix deposition and fluid accumulation (Plodinec et al., 2012; Stylianopoulos et al., 2012; Nagelkerke et al., 2015; Brauchle et al., 2018). The altered mechanical micro-environment in tumors affects multiple processes including cell invasion (Levental et al., 2009; Acerbi et al., 2015) and phenotypic state (Wei et al., 2015), but likely also impacts the cell division process. We have previously proposed that completing cell division in such an altered mechanical environment presents a challenge that could be overcome by enhanced mitotic rounding in cancer cells (Matthews and Baum, 2012). This is supported by the frequent observation of highly rounded mitotic cells in cancer samples and tumoroids (Figure 3C). In addition, some of the key genes required for mitotic rounding, including Ect2 and Ezrin are frequently over-expressed in human cancers (Bruce et al., 2007; Fields and Justilien, 2010).

Several recent studies by us and others have revealed that molecular changes that occur during oncogenesis can alter the ability of cells to apply force at mitosis. Work by Hosseini and colleagues reports that epithelial-mesenchymal transition (EMT), a phenotypic transformation commonly found in many epithelial-derived cancers and frequently associated with invasion and metastasis (Yang et al., 2020) alters the mechanical properties of the mitotic cortex and the ability of cells to round up in stiff 3D environments (Hosseini et al., 2019). The observed changes in cortex mechanics were associated with changes in RhoA and Rac1 activity induced by EMT (Hosseini et al., 2019). Since surrounding interphase cells also become more compliant when undergoing an EMT, mitotic rounding may be further enhanced. There is also evidence that loss of E-cadherin expression, a change associated with EMT can directly alter mitotic cell mechanics: mitotic epithelial cells in a monolayer were found to have increased cortical contractility and elasticity after E-cadherin knockout, as measured by AFM (Rhys et al., 2018). Vimentin, which is induced by EMT and has recently been found to regulate cortical tension in mitosis (Duarte et al., 2019; Serres et al., 2020), may also contribute. Thus, there are likely multiple pathways by which EMT affects the ability of cells to exert force during mitotic rounding that may play critical roles in the ability of cells to proliferate and invade out of tumors.

Most cancers are driven by mutations in a handful of oncogenes, and we recently addressed the role of one such oncogene, Ras, in regulating mitotic mechanics (Matthews et al., 2020). We found that Ras activation led to an acceleration of mitotic rounding and increased cortical stiffness at mitotic entry. These changes were observed only a few hours after Ras activation, pointing to an early, direct mechanism by which oncogenic mutations affect mitotic mechanics that requires downstream MEK/ERK signaling and acto-myosin contractility (Matthews et al., 2020). Activation of the oncogenic Ras/MEK/ERK signaling pathway has been shown to modulate the actin cytoskeleton during oncogenesis to promote cell motility and invasion (Choi and Helfman, 2014; Logue et al., 2015; Mendoza et al., 2015; Schäfer et al., 2016) and similar mechanisms may be in play during mitotic stiffening. These changes in mechanics following oncogene activation have consequences. A function for Ras-induced mitotic stiffening was revealed when cells were placed in confinement under a stiff gel: Ras-activated cells were able to stiffen enough at mitosis to deform the gel and make space for functional spindle assembly, while normal cells suffered multiple mitotic defects (Matthews et al., 2020). This suggests that genetic mutations that drive cancer progression such as Ras-activation can promote accurate cell division in stiff or confined environments. Another example of mitotic stiffening supporting accurate cell division was observed in the centrosome clustering in cells with centrosome duplication, an abnormality frequently seen in cancer cells (Godinho and Pellman, 2014). Cells with increased cortical elasticity following E-cadherin knockdown were more efficient at clustering supernumerary centrosomes, facilitating bipolar spindle formation and division (Rhys et al., 2018). The idea that enhanced mitotic rounding can minimize mitotic errors in cancer is somewhat paradoxical, as cancers are often characterized by a high degree of aneuploidy, generated through errors in chromosome segregation in mitosis (Sansregret and Swanton, 2017; Nelson et al., 2020). However, some of the mitotic errors observed when mitotic rounding is compromised such as tripolar division are unlikely to result in viable daughter cells. Thus, the ability of cancer cells to apply force to divide in stiff environments may allow continued cell proliferation and survival, while at the same time allowing introduction of occasional chromosome segregation defects, contributing to the development of aneuploidy in the longer term.

Finally, as well as promoting faithful spindle formation in mitosis, there may be mechanisms whereby mitotic rounding and stiffening in cancer contributes to disease progression. There have been several reports of mitotic rounding driving changes in tissue structure. Mitotic rounding of individual cells within a monolayer of breast cancer cells triggers a 2D to 3D tissue transformation (Lee et al., 2018). Individual mitotic rounded cells are also able to bud out of epithelial acini in 3D culture, although only when the surrounding interphase cells are stiffened due to centrosomal aberrations, hinting at a role for mitosis in cancer cell dissemination (Ganier et al., 2018). Mitotic rounding may even affect the response to therapy: mitotic arrest following treatment with the chemotherapy agent paclitaxel led to a prolonged cell rounding that resulted in mitotic cells being targeted for engulfment by entosis (Durgan et al., 2017). These intriguing studies hint at a role for changes in cell shape, specifically in mitosis, in promoting a variety of other cancer cell behaviors including invasion and dissemination.

## Outlook

A wealth of work using different techniques has demonstrated that cells change their mechanical properties when they enter mitosis. In recent years, we are beginning to form an understanding of how this happens in single cells, downstream of mitotic kinases, through the formation of a high tension acto-myosin cortex and an increase in intracellular pressure. However, there remain many unanswered questions about the molecular regulation of mitotic rounding. For example, how molecular changes to the architecture of actin networks result in a mitotic cortex, which is both stiffer and thinner than in interphase. Although there is clear evidence that water enters the cell to increase intracellular pressure, the mechanisms that control the underlying changes in osmolarity are as yet unknown. The function of mitotic stiffening has been revealed by confinement studies using single cells. By exerting pressure against their environment, cells create space for spindle formation and faithful cell division. However, mitotic stiffening is likely to be of greater importance for cells dividing in tissues, but little is known about the extent of mechanical changes during mitosis *in vivo*, nor how acto-myosin contractility is regulated in a co-ordinated way to allow mitotic rounding while ensuring tissue integrity by maintaining cell/cell adhesion. Finally, several recent studies have revealed that changes associated with cancer, such as oncogene activation or EMT, can directly impact mitotic mechanics hinting at a role for mitotic rounding in facilitating cell division in tumors. Studying this further will rely on non-invasive techniques that allow the mechanical properties of living tissues under normal and diseased conditions to be interrogated.

## Author Contributions

AT, BB, and HM conceived the ideas, discussed, and edited the manuscript. AT and HM wrote the manuscript and made the figures. All authors contributed to the article and approved the submitted version.

## Conflict of Interest

The authors declare that the research was conducted in the absence of any commercial or financial relationships that could be construed as a potential conflict of interest.

## Figures and Tables

**Table:** 

Surface/cortical tension	Surface tension: Tension resulting from forces acting in the plane of the surface of a liquid tending to minimize its surface area. In the context of a rounded cell, both cortical tension and membrane tension contribute to the cell’s effective surface tension, with cortical tension dominating (Tinevez et al., 2009; Fischer-Friedrich et al., 2014). Contractile elements of the cortical acto-myosin cytoskeleton are cause of the tension generation in the cortex.
Intracellular pressure	In a fluid-filled spherical object like a cell (or balloon) at steady-state, the pressure difference between the external and internal pressure is related to surface tension and curvature according to Laplace law (see below). Tension-build up in the cortex results in an increase in (hydrostatic) pressure. In the cell, intracellular pressure changes can also arise from water flux across the membrane (due to ion fluxes across the membrane).
Cortical stiffness	Resistance of the cell cortex to bending under force. This depends on its viscoelastic properties, cortical tension, geometry and experimental settings. Also referred to as cortex rigidity.
Laplace law	Describes the relationship between surface tension T, the pressure difference between the inside and outside ΔP and the radius of curvature R (e.g., for a sphere) in a fluid-filled spherical object (such as a rounded cell) (Yeung and Evans, 1989). 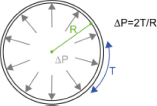
Stress	Defined as force (F) per unit area (A). The type of stress depends on how forces act, e.g., normal stress (*σ* = F/A) (perpendicular on surface, either compressive or tensile) or shear stress *(τ* = F/A) (parallel to surface). 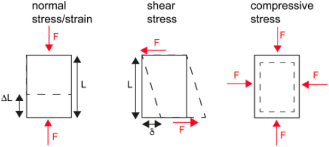
Strain	Ratio of deformation in direction of the force (ΔL) relative to initial length (L), e.g., normal strain *ε* = ΔL/L or shear strain *γ* = δ/L (both dimensionless).
Elasticity	Ability of a material to resist a deforming force and to return to its initial shape upon force removal.
Elastic modulus	Ratio of stress and strain, quantifies the resistance of an object to elastic deformation upon stress application. There are three moduli defined depending on force direction application: the Youngs modulus E, the shear modulus G, and the compression/bulk modulus K (see below). These moduli are related for a linear-elastic isotropic material as: E = 2G(1+ν) = 3K(1-2ν) = 9KG/(3K+G) (ν: Poisson ratio); two of them are sufficient to capture the elastic behavior of a material (Landau and Lifshitz, 1986).
Young’s modulus E	Relates normal stress σ and normal strain ε (or how much stress is applied to obtain a certain level of strain), e.g., for simple case of uniaxial deformation *σ* = Eε (Hook’s law for one-dimensional case).
Shear modulus G	Relates shear stress τ and shear strain γ, τ = Gγ.
Bulk/compression modulus	Describes how much change of pressure is needed for a certain volume change (volumetric elasticity).
Viscoelasticity	Property of a material displaying both elastic and viscous mechanical behavior. Viscoelastic materials display a frequency dependent stress-strain response. Therefore, the timescale at which they are probed matters.
Complex, storage & loss modulus	The complex moduli G^∗^ or E^∗^ can be defined for viscoelastic materials (analogous to the elastic moduli, Shear and Young’s moduli, for elastic materials). G^*^ = G′+iG^”^ (or E^∗^ = E′+iE″, respectively) with its real part, the storage modulus G′ (E′) and its imaginary part, the loss modulus G”(E”). The storage modulus relates to the ability of a material to store energy elastically, while the loss modulus is related to the ability of a material to dissipate energy. Can be measured in oscillatory measurements, as in phase (storage) and out of phase components (loss) of the stress to strain response (Jacobs et al., 2012).
